# Lipid Accumulation and Chronic Kidney Disease

**DOI:** 10.3390/nu11040722

**Published:** 2019-03-28

**Authors:** Zhibo Gai, Tianqi Wang, Michele Visentin, Gerd A. Kullak-Ublick, Xianjun Fu, Zhenguo Wang

**Affiliations:** 1 Key Laboratory of Traditional Chinese Medicine for Classical Theory, Ministry of Education, Shandong University of Traditional Chinese Medicine, Jinan 250355, China; zhibo.gai@usz.ch; 2 Institute for Literature and Culture of Chinese Medicine, Shandong University of Traditional Chinese Medicine, Jinan 250355, China; wangtianqi9292@gmail.com; 3 Department of Clinical Pharmacology and Toxicology, University Hospital Zurich, 8006 Zurich, Switzerland; michele.visentin@usz.ch (M.V.); gerd.kullak@usz.ch (G.A.K.-U.); 4 Mechanistic Safety, CMO & Patient Safety, Global Drug Development, Novartis Pharma, 4056 Basel, Switzerland

**Keywords:** lipid accumulation, blood lipids, metabolic disease, chronic kidney disease, potential therapeutic strategy

## Abstract

Obesity and hyperlipidemia are the most prevalent independent risk factors of chronic kidney disease (CKD), suggesting that lipid accumulation in the renal parenchyma is detrimental to renal function. Non-esterified fatty acids (also known as free fatty acids, FFA) are especially harmful to the kidneys. A concerted, increased FFA uptake due to high fat diets, overexpression of fatty acid uptake systems such as the CD36 scavenger receptor and the fatty acid transport proteins, and a reduced β-oxidation rate underlie the intracellular lipid accumulation in non-adipose tissues. FFAs in excess can damage podocytes, proximal tubular epithelial cells and the tubulointerstitial tissue through various mechanisms, in particular by boosting the production of reactive oxygen species (ROS) and lipid peroxidation, promoting mitochondrial damage and tissue inflammation, which result in glomerular and tubular lesions. Not all lipids are bad for the kidneys: polyunsaturated fatty acids (PUFA) such as eicosapentaenoic acid (EPA) and docosahexaenoic acid (DHA) seem to help lag the progression of chronic kidney disease (CKD). Lifestyle interventions, especially dietary adjustments, and lipid-lowering drugs can contribute to improve the clinical outcome of patients with CKD.

## 1. Introduction

Chronic kidney disease (CKD) is defined by a reduced glomerular filtration rate (GFR) to less than 60 mL/min per 1.73 m^2^ and/or alteration of kidney damage marker values (e.g., proteinuria) of at least 3 months duration, regardless of the underlying cause [1]. The latest data from the Global Burden of Disease (GBD), Injuries, and Risk Factors study reported that, worldwide, CKD-related morbidity and mortality are rising—a trend that also carries a substantial financial burden [2]. Currently, the costs for the treatment and care of patients with CKD outrank those for other common diseases [3].

The etiopathogenesis of CKD is still unclear, yet a plethora of risk factors has been identified. Among these, obesity and hyperlipidemia are becoming more and more prevalent. In particular, obesity seems to be strictly interrelated with CKD [4]. Consistently, CKD shares a number of key features with the obesity-related glomerulopathy (ORG), which is characterized by glomerular enlargement, focal and segmental sclerosis (FSGS), damage of the peritubular capillaries, and retrograde glomerular changes [5,6,7]. This review systematically describes the link between lipids and CKD development and progression from epidemiological, clinical and pathophysiological angles, and it provides new insights into the potential lipid-related therapeutic approaches for patients with CKD.

## 2. Epidemiological Studies

People with elevated body mass index (BMI) have a higher risk of developing CKD compared to individuals with a BMI in the normal range [2]. It has been estimated that for each BMI unit in excess, the risk of CKD augments by 5% [2]. Obesity is now considered an independent risk factor for CKD onset [8]. Additionally, obesity is also a risk factor for hypertension and cardiovascular diseases, which are themselves risk factors for CKD [9]. Obesity-related events have a detrimental influence on renal function and have been linked to end-stage renal disease [10]. The prevalent hypothesis is that individuals with a high BMI (≥25 kg/m^2^) experience substantial changes in renal hemodynamics (GFR, renal plasma flow and filtration fraction), perhaps as a result of excessive sodium intake, increasing their risk of long-term kidney damage [11,12]. However, other abnormalities, often co-existing in obese individuals, such as insulin resistance and hyperlipidemia may contribute to the renal damage. In addition, patients with one or more of these factors are more prone to develop CKD as they gain weight [4,13]. A survey of 5897 hypertensive patients showed that after the adjustment for covariates such as diabetes, both baseline overweight (Odds Ratio: 1.21, 95% CI: 1.05–1.41) and obesity (Odds Ratio: 1.40, 95% CI, 1.20–1.63) were associated with CKD [4]. The visceral adiposity index and the lipid accumulation product index positively correlated with CKD [14,15].

In the early 1990s, large-scale and case studies proposed a link between hyperlipidemia and CKD. After 10 years of follow-up, it was found that the hazard ratio of proteinuria increased in both genders in CKD patients with hypercholesterolemia, low high-density lipoprotein levels (HDL), and high triglyceride levels [16]. A cross-sectional community-based Japanese study associated hyperlipoproteinemia with low estimated GFR [17]. In a meta-analysis by Weiner, 90% of the patients with CKD displayed total cholesterol levels higher than 240 mg/dL, and more than 80% of the patients with CKD who were not treated with hemodialysis, had low-density lipoprotein (LDL) levels above 130 mg/dL [18]. This finding was confirmed by two following studies [19,20]. Besides the cholesterol and triglyceride (esterified fatty acids) levels, the excess of non-esterified fatty acids (also known as free fatty acids, NEFAs or FFAs) and long polyunsaturated complex lipids can aggravate CKD patient’s condition [21]. There is enough epidemiological evidence to hypothesize a causative link between lipid accumulation and the development and progression of CKD.

## 3. The Mechanism of Lipid Transport and Metabolism in the Kidneys

**Figure 1:**
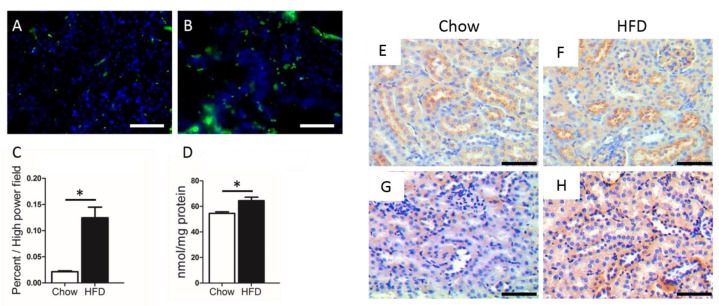
Representative images of BODIPY staining (**A**,**B**) in kidney sections from chow diet (**A**) and high fat diet (HFD) (**B**) mice. Scale bar = 50 μm. Analysis of BODIPY staining in different kidney sections (**C**). Total cholesterol content in the kidney from different groups (**D**). *n* = 6 mice/group, * *p* < 0.05. Representative images of immunostaining for FATP4 (**E** and **F**) and 4-HNE (**G** and **H**) in kidney sections from chow and HFD mice. Scale bar = 50 μm.

Hyperlipidemia is caused by an abnormal lipid intake and/or metabolism, resulting in hypercholesterolemia, hypertriglyceridemia, or both (combined hyperlipidemia) [22]. When energy intake gradually exceeds the body’s ability to store fat in adipose tissue (white adipose tissue), circulating lipids from various sources spill over into non-adipose tissue, such as muscle, liver, kidneys, and pancreas; this phenomenon is called “ectopic lipid accumulation” [23]. In the kidneys, lipids can sediment in virtually all cell types, from mesangial cells to podocytes and proximal tubule epithelial cells. For instance, in mice fed a high-fat diet (HFD), the high systemic levels of neutral lipids (e.g., cholesterol) and phospholipids lead to “fatty” proximal tubules (Figure 1, own data) [24]. Moreover, lipid levels continue to increase with the progression of kidney lesions [25,26].

### 3.1. Lipid Uptake by CD36 in the Kidney

CD36 is a multifunctional transmembrane glycoprotein that mediates the uptake of oxidized LDL (ox-LDL) [27]. Loss of function mutations in the *CD36* gene cause abnormal levels of plasma fatty acids (FA) and triglycerides in mice [28]. CD36 is the main FA uptake system in the kidneys and appears to play a central role in CKD development and progression. Higher renal levels of CD36 were found in patients and animals with kidney damage [29]. Kidney CD36 expression levels were higher in CKD patients with diabetic nephropathy and were associated with altered renal lipid accumulation [20,30]. Chronic inflammation, a feature of obesity, is known to induce CD36 expression, which can aggravate kidney damage and accelerate the disease progression [30]. Mice lacking CD36 displayed lower renal lipid accumulation and were less susceptible to kidney damage and related complications in comparison with the respective wild-type animals [30].

**Figure 2:**
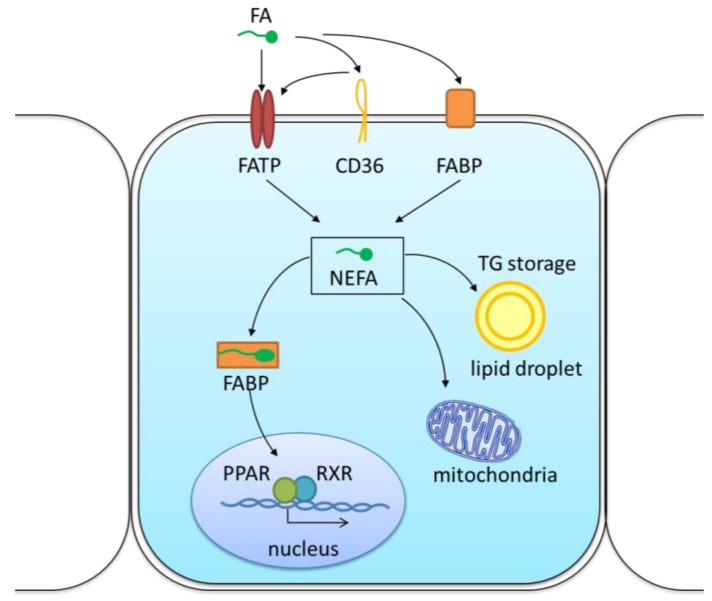
Schematic representation of fatty acids cellular uptake in the kidneys. FA transport across the plasma membrane occurs mainly by protein-mediated mechanisms either with CD36 or with FATPs. In the cells, FAs bind to different FABPs with respect to the subcellular localization and have multiple functions in energy generation and storage, membrane synthesis and activation of nuclear transcription factors like PPAR/RXR. NEFA, non-esterified fatty acid; FATP, fatty acid transport protein; FABP, fatty acid-binding protein; PPAR, peroxisome proliferator activated receptor; RXR, retinoid X receptor; TG, triglyceride.

CD36 is highly expressed in renal proximal and distal tubular epithelial cells, podocytes, mesangial cells, microvascular endothelial cells, and interstitial macrophages (Figure 2) [30,31,32,33,34]. In macrophages, CD36 levels were shown to correlate with the intracellular levels of ox-LDL [35]. Ox-LDL are metabolized to 9-hydroxyoctadecenoic acid and 13-octadecenoic acid, which activate the peroxisome proliferator-activated receptor γ (PPARγ), a transcription factor involved in adipogenesis. PPARγ activation also institutes a positive feedback by *trans*-activating the *CD36* gene promoter, thus increasing CD36 expression [36]. In podocytes, CD36-mediated palmitic acid uptake resulted in a dose-dependent increase in reactive oxygen species (ROS) levels, mitochondrial membrane potential depolarization, ATP depletion, and activation of the apoptotic pathways [32,37,38]. Additionally, it was shown in animals that FAs can trigger apoptosis of renal tubular epithelial cells and podocytes by inducing the expression of thrombospondin 1, a ligand of CD36 [39,40].

Possessing a positively charged binding pocket, CD36 preferentially binds negatively charged ligands. Besides ox-LDL, advanced oxidation protein products (AOPPs), and advanced glycation end products (AGEs), which are known to promote inflammation and atherosclerosis, are recognized by CD36 as substrates [41]. Phosphorylation, glycosylation, and palmitoylation of the extracellular site of CD36 can affect FA uptake rate as well, nevertheless whether the post-translational modification pattern of CD36 is affected by obesity and hyperlipidemia has not been studied yet [42,43,44,45,46]. CD36 can also interact with the Na^+^/K^+^-ATPase and promote inflammation and oxidative stress upon binding to a variety of circulating ligands (e.g., cardiotonic steroids). Several of these ligands are increased by high fat diet, potentiating this CD36/Na^+^/K^+^-ATPase-dependent inflammatory paracrine loop between proximal tubule cells and macrophages, and thereby facilitating the development of chronic inflammation, oxidant stress, and fibrosis underlying the renal dysfunction [30].

### 3.2. Lipid Uptake by Other Transporters

Several fatty acid transport proteins (FATPs, SLC27A1-6) facilitate FFA cellular uptake (Figure 2) [47,48,49]. Recent studies indicate that an abnormal FATP-mediated transport directly affects lipid homeostasis, which, in turn, can trigger obesity, dyslipidemia, diabetes, and other diseases [50,51]. Figure 1 shows that FATP4 expression was higher in mice fed a HFD (own data). These animals also displayed a higher degree of lipid peroxidation (4-Hydroxynonenal, 4-HNE, own data).

While knowledge of the physiological role of FATP comes mainly from studies on adipose tissues, some members of this family, specifically FATP1 (*SLC27A1*), FATP2 (*SLC27A2*), and FATP4 (*SLC27A4*), are also highly expressed in the kidneys [48]. FATP1 is a peri-membranous protein, which are trafficked to the cell surface in response to insulin [52]. The function of FATP1 has been mainly characterized in the adipose tissue, in which the expression is induced when preadipocytes differentiate into adipocytes [49]. FATP2 has been shown in mice to regulate lipid synthesis in the kidney—the repression of FATP2 led to the decrease of acyl-CoA synthetase activity in the kidney, resulting in a reduced lipid synthesis in the endoplasmic reticulum (ER) [53,54]. The transport of FFAs into the ER is mainly mediated by FATP4. FATP4 functions as an acyl-CoA synthetase with preference for very long-chain fatty acids [55]. FATP4 expression and polymorphisms have been associated with human insulin resistance and obesity [56].

Other membrane proteins that can facilitate FA uptake are the plasma membrane fatty acid-binding protein (FABPpm) (Figure 2) [57]. FABPs are members of the lipid-binding protein superfamily that preferentially recognize long-chain fatty acids as substrates. In spite of the names, the epidermal FABP (E-FABP), the liver-type FABP (L-FABP) and the heart-type FABP (H-FABP) are those expressed in the kidney and appear to be physiologically redundant [58]. In fact, in animals, the deletion of the *L-FABP* gene resulted in increased expression of E-FABP and cholesterol accumulation [59]. FABPs also facilitate the polyunsaturated FA-induced transactivation of PPARα and PPARγ via direct interaction with the nuclear receptor’s ligand binding domain [60,61,62,63,64]. FAs also induce FABP expression [65]. In the rat kidney disease model, high levels of L-FABP promoted the proliferation of rat tubular epithelial cells and aggravated interstitial inflammation [66].

### 3.3. Regulation of Lipogenesis in the Kidney

The regulation of lipogenesis is mediated by a number of transcriptional factors such as the CCAAT/enhancer-binding protein α (C/EBPα), PPARγ, sterol regulatory element-binding protein 1 (SREBP-1) and farnesoid X receptor (FXR) [67]. The C/EBPα fine-tunes glucose homeostasis by indirectly regulating insulin signaling but also participates in the regulation of triglycerides synthesis [68]. In diabetic rats, the expression of SREBP-1, induced by high glucose diet, induced fatty acid synthesis [69]. It has been shown that the hepatic lipid content raised as a result of the repression of several enzymes involved in mitochondrial β-oxidation and the overexpression of carbohydrate-responsive element-binding proteins and their target genes, which are the main regulators fatty acids synthesis, suggesting that lipogenesis regulation occurs mainly by modulating fatty acid β-oxidation in the mitochondria [70]. This might also happen in the kidney, albeit no experimental evidence is reported. In a recent work, the adipose triglyceride lipase (ATGL), which catalyzes the initial step in triglyceride hydrolysis in adipocyte, was also found to affect renal lipid content. ATGL-deficient mice exhibit significantly higher triglycerides levels in the kidney in comparison with the respective wild-type animals, accompanied by proteinuria [71]. Other potential mechanisms of lipid accumulation in the kidneys may be related to the dysregulation of multipotent mesenchymal progenitor cells (MMPCs). In fact, in certain conditions MMPCs can represent an additional pre-fat cell population. MMPC differentiation disorder has been hypothesized to be related to the uremic environment secondary to the low renal performance [72].

## 4. Lipid-Induced Renal Damage

Most studies have focused on the lipid nephrotoxicity hypothesis based on Moorhead’s work [26]. Scholars who support this hypothesis believe that hyperlipidemia can lead to inflammation, ROS production and endogenous electrical stress. There are pieces of evidence that renal lipid accumulation can cause structural and functional changes in mesangial cells, podocytes and proximal tubule cells, which all contribute to the nephron function [73]. However, to date, there is only limited evidence for an association of lipid accumulation with kidney disease.

One of the main sites of renal lipid accumulation is the renal proximal tubule cells. High levels of albumin-bound long-chain saturated fatty acids were shown to promote the progression of renal tubular damage and interstitial fibrosis; excess of Ox-HDL induced pro-inflammatory pathways, including tumor necrosis factor-alpha (TNF-α), CC motif chemokine 2 and interleukin-6 (IL-6), and increased the production of ROS [30,74,75]. Recent animal studies have shown that deletion of the gene encoding for the biliverdin reductase A (BVRA) caused lipid accumulation and toxicity in mouse proximal tubular cells, and this may be related to impaired mitochondrial respiration and β-oxidation [20]. BVRA was also shown to protect the kidney by modulating insulin signaling [76]. Renal tubule genome-wide analysis showed that in both mice and human renal interstitial fibrosis models, there was a lower expression of numerous important mitochondrial enzymes, and fatty acid oxidation adjustment factors, such as PPARα and PPARγ, resulting in increased lipid deposition in cells [77]. Accumulation of triglycerides was also found to accelerate renal tubule fibrosis [26]. Proximal tubule cells seem to be more susceptible to lipid toxicity than the other cell population of the kidneys, perhaps because they require higher energy expenditure, which can only be supplied by oxidative phosphorylation at the mitochondrial level. Hence, lipid-induced mitochondrial damage might be particularly catastrophic for proximal tubule cells [78,79].

Lipid-induced toxicity also contributes to the development of glomerulosclerosis. Kidneys from rats fed a HFD for 32 weeks were characterized by chronic inflammation, high ROS level, and fibrosis in the glomeruli [34]. In patients with obesity-related proteinuria, renal biopsies showed glomerular hypertrophy and FSGS lesions, perhaps due to inhibition of the AMPK/PGC1α pathway secondary to the accumulation of sphingomyelin in mesangial cells [80,81]. Lipid-induced glomerulosclerosis may also be the result of the concerted activation of SREBP-1, transforming growth factor 1 (TGF-1), vascular endothelial growth factor (VEGF), and inflammatory pathways [69,73]. Rats on HFD with chronic inflammation developed severe renal degeneration and glomerular damage, indicating that the aggravation of obesity may itself exacerbate existing kidney damage, perhaps due to the overexpression of CD36, TNFα, IL-6, and monocyte chemotactic protein-1 (MCP-1) during inflammation, which results in the thickening of the glomerular basement membrane, extracellular matrix, glomerulosclerosis [34]. In particular, lipid accumulation can initiate ER stress to enhance TNFα or IL-6 in HMC and HK2 cells, resulting in the increased production of ROS and direct toxic effects on the kidney [34]. Elevated levels of ROS markers will aggravate endothelial dysfunction and vascular disease in CKD and cause an increase in uremic toxins [82]. Podocytes represent another type of cells whose damage contributes to glomerulosclerosis and albuminuria in CKD. Podocytes adhere to the outside of the glomerular basement membrane (GBM), together with the vascular endothelial cells and the glomerular basement membrane, and constitute a glomerular hemofiltration barrier. Current findings in mice provide evidence that CD36 can interact with thrombospondin-1 (TSP-1) in podocytes, and triggers apoptosis [39]. Podocyte dysfunction and apoptotic rate were reduced in obese mice lacking TSP-1 compared to the wild type obese [40]. In the ATGL-deficient mouse model, which is characterized by renal lipid accumulation and proteinuria, the rate of apoptotic podocytes is elevated in comparison with that of podocytes from wild type animals. The transient silencing of ATGL could promote podocyte apoptosis, which might be the result of increase of ROS, actin rearrangement and foot process fusion [71].

## 5. Treatment

### 5.1. Bile Acid Receptor Agonists

The farnesoid x receptor (FXR) is a bile acid nuclear receptor abundant in the intestine and in the liver and recently found highly expressed also in the kidney [83]. Because it is a key modulator of bile acid and lipid metabolism, and limits the production of ROS, pro-inflammatory cytokines and pro-fibrotic growth factors, FXR represents a potential target for the treatment of CKD [83,84]. Studies have shown that in HFD-induced obese mice, the treatment with a FXR agonist reduced plasma total cholesterol concentration by downregulating the expression level of SREBP-1 in different tissues, including the kidneys [83]. The treatment of obese mice with the semi-synthetic FXR agonist obeticholic acid (OCA) reduced the degree of glomerular sclerosis and tubulointerstitial injury by improving mitochondrial function and promoting FA oxidation, which, in turn, reduced mitochondrial stress and the ER stress [84]. In another study, administration of OCA to mice with ischemia/reperfusion kidney damage could prevent early-stage renal damage by reducing monocyte infiltration and oxidative stress, thereby protecting the kidney from CKD on the long term [85]. In addition, a recent study on non-alcoholic fatty liver disease has shown that the anti-inflammatory effect of FXR activation was the result of a switch in arachidonic acid metabolism: from pro-inflammatory (leukotrienes) to anti-inflammatory (epoxyeicosatrienoic acids) [86]. Pharmacological activation of FXR appears to be safe and can represent a valid treatment option for the continuously increasing number of overweight patients with CKD [86,87].

The G protein-coupled bile acid receptor GPBAR1 (TGR5) is a member of the G protein-coupled receptor (GPCR) superfamily and is a bile acid receptor, which plays a key role in the regulation of energy consumption and glucose homeostasis. Because its expression and activity were found reduced in the kidneys of obese and diabetic individuals, TGR5 appears to be an intriguing target for the treatment of metabolic diseases such as type 2 diabetes and obesity [88,89]. Mice fed a HFD had decreased blood glucose and insulin levels after treatment with a natural TGR5 agonist (a triterpene, i.e., oleanolic acid contained in olive leaves), and prevented the animals fed a HFD from gaining weight [88]. In another animal study, the use of the TGR5 agonist INT-777 could reduce urinary albumin excretion, glomerular mesangial expansion, and accumulation of extracellular matrix proteins and plasma triglycerides in obese mice with kidney dysfunction [90]. The activation of TGR5 prevented the progression of nephropathy in diabetic and obese animals by inducing mitochondrial biogenesis and preventing lipid accumulation and oxidative stress [91].

### 5.2. PPAR Agonists

PPARα is a member of the ligand-activated transcription factor nuclear receptor superfamily and it is primarily involved in fatty acid β-oxidation and lipolysis [92,93]. PPAR*α* is expressed both in glomerular and tubular cells. Fenofibrate, a potent PPAR*α* agonist, was shown to reduce systemic triglyceride levels, renal oxidative stress, proteinuria and glomerular sclerosis with an overall improvement of the renal function in mice fed a HFD [94,95].

PPARγ regulates lipogenesis and is abundantly expressed in adipocytes and immune cells [96]. PPARγ activation increases insulin sensitivity, which in turn lowers serum glucose concentrations [97,98]. Because endogenous ligands such as long chain polyunsaturated fatty acids and eicosanoid derivatives can activate PPARγ, it is conceivable that PPARγ agonists can be used for reducing lipid systemic levels and renal lipid accumulation in obese patients with CKD [99,100]. Thiazolidinediones-like PPARγ agonists, such as rosiglitazone, significantly reduced blood glucose and triglyceride concentrations, and insulin resistance in diabetic (db/db) mice [101]. Insulin resistance is also a common feature in patients with CKD. In a randomized, double blind, placebo-controlled study, 8-weeks treatment of rosiglitazone in CKD subjects lowered the homoeostasis model assessment (HOMA) score and high-sensitivity C-reactive protein (hs-CRP), indicating an increased insulin sensitivity in patients with CKD treated with rosiglitazone [102]. The inhibition of the Wnt signaling-mediated fibrogenesis may represent another mechanism of kidney protection by PPARγ activation [103,104]. Moreover, PPARγ also participates in the regulation of antioxidant production, including superoxide dismutase 2 and glutathione peroxidase 1, which play important roles in metabolic disorders and progression of CKD [105].

### 5.3. C/EBPα and C/EBPβ Inhibition

The C/EBPα is a major transcription factor in lipogenesis and it is involved in the stimulation of the mononuclear phagocyte system, which increases oxidative stress and inflammation [106,107,108,109,110]. Interstitial fibrosis is prominent in the kidneys of patients with end-stage CKD [111]. Fibrosis lesion degree, infiltration degree, and fibrosis proportion were found to positively correlate with C/EBPα expression [112]. Thus, lipid accumulation may contribute to the activation of the mononuclear phagocyte system and inflammation. Macrophage phagocytes can also oxidize lipids and transform into foam cells, which, in turn, recruit more macrophages that aggravate the lesions, lipid deposition, endothelial cell function, and vascular smooth muscle cell (VSMC) proliferation [113].

C/EBPβ is an essential factor in initiating several transcriptional cascades [114]. Knocking down C/EBPβ in 3T3-L1 preadipocytes could block adipogenesis, whereas C/EBPβ overexpression was sufficient to induce the differentiation of 3T3-L1 into adipocytes [115,116,117]. The C/EBPβ is also involved in mitotic clonal expansion (MCE), an essential process for terminal adipocyte differentiation. In C/EBPβ(^−/−^) mice, embryonic fibroblasts were not able to initiate the MCE process, hence could not differentiate into adipocytes [114,116]. These findings suggest that C/EBPβ can have an effect on renal lipid accumulation in patients with CKD, and the data reported here appear to support the assumption that C/EBPβ can be used as the therapeutic target of CKD.

### 5.4. CD36 Antagonists

As mentioned above, there is a strong link between CD36 and lipid accumulation in renal tissues. Therefore, CD36 is an intriguing target in CKD treatment. A recent study has shown that in nephrectomized mice, the use of the CD36 antagonist 5A peptide (an apoA-I-mimetic peptide) could reduce the degree of renal inflammation and tubulointerstitial fibrosis and slow down the progression of CKD [118]. Cyclic azapeptides could also be used as a peptidomimetic therapy for CD36 inhibition [119]. It has also been shown that azapeptide EP80317 improved post-ischaemic damage caused by myocardial infarction/myocardial infarction by (i) inhibiting CD36 activity and (ii) transiently reducing peripheral lipolysis, thus preventing the movement of FAs from adipose tissue into the blood and other tissues [120]. The synthetic amphipathic helical peptides ELK (ELK-SAHPs) that target CD36 could also inhibit CD36 expression, and ameliorated lung inflammation and function [121]. Theoretically, blocking major receptors/transporters involved in renal fatty acid absorption can represent an innovative treatment for patients with CKD. However, response to the treatment and potential side effects of this approach need further evidence from clinical data.

### 5.5. SREBP-1 Inhibitors

As discussed above, SREBPs are transcription factors that govern cholesterol biosynthesis and transport, and FA biosynthesis. In animals, the renal expression of sterol regulatory factor binding proteins (SREBP-1 and SREBP-2) in the HFD group was found significantly increased as compared with that of lean mice, causing the accumulation of cholesterol and triglycerides in the renal parenchyma, and the induction of pro-fibrotic genes such as type IV collagen and fibronectin [122]. Insulin-treated diabetic rats showed reduced SREBP-1 expression and triglyceride accumulation, suggesting that the treatment of diabetic rats with insulin could inhibit the expression of SREBP-1 in the kidneys and reduce the accumulation of triglycerides [123]. Reduction of SREBP-1 expression using ENOblock reduced the accumulation of lipids in the kidneys of type 2 diabetic mice and reduced the degree of fibrosis and inflammation [124].

### 5.6. Other Targets

Another signaling pathway involved in adipogenesis that represents an intriguing drug target for the treatment of CKD is the Janus-activated kinase/signal transducer and activator of transcription (JAK-STAT) pathway. In fact, in both patients and animals with CKD, the overexpression and/or hyperactivation of the JAK-STAT signaling pathway appeared to worsen the kidney injury [125]. While no studies are available on the therapeutic role of JAK inhibitors in patients with CKD, it is conceivable that efficient inhibition of this pathway may contribute to minimize the renal dysfunction. JAK inhibitors are already safely administered for the treatment of a spectrum of hematological diseases (e.g., leukemia) and autoimmune disorders (e.g., rheumatoid arthritis and psoriasis) [126,127,128,129].

The role of adrenocortical hormones in kidney and adipose tissues is well characterized. Indeed, glucocorticoids and aldosterone-based mineralocorticoid drugs are effective in the treatment of CKD, especially for their anti-inflammatory effect [130]. Nonetheless, methylprednisolone has also been shown to inhibit the Wnt/b-catenin signaling pathway in murine subcutaneous and visceral adipose tissue and to increase the expression of ATGL and hormone sensitive lipase (HSL) in subcutaneous adipose tissue. The induction of ATGL and HSL ultimately reduced the animal body fat and weight [131]. In addition, dexamethasone selectively inhibits the glucocorticoid receptor, thereby inhibiting the expression of pro-inflammatory adipokines [123].

Statins are competitive inhibitors of the HMG-CoA reductase, the rate-limiting enzyme of the mevalonate pathway, and are widely used to reduce total cholesterol, LDL, triglycerides, and HDL. The cardiovascular and renal protective effect of lowering cholesterol in patients with kidney dysfunction is still controversial. Fluvastatin did not reduce rates of coronary intervention procedures or mortality in renal transplant recipients [132]. Conversely, the double-blind SHARP study (Study of Heart and Renal Protection) on over nine thousand patients with CKD found that simvastatin in combination with ezetimibe (inhibitor of cholesterol intestinal absorption) reduced total cholesterol, LDL and the incidence of major atherosclerotic events, but no benefit to the kidney function was observed [133]. Finally, a recent retrospective cohort study by Hu et al. showed that patients with CKD stage 3B-5 taking statins have a significantly lower proteinuria as compared with those patients who were not taking statins (Odds Ratio: 0.68, 95% CI 0.48–0.95) [134]. A summary of the most relevant studies on lipid-lowering drugs for the treatment of CKD is reported in Table 1.

### 5.7. Non-Targeted Therapies

Nearly 80% of the world’s population, especially in developing countries, rely on botanical drugs as part of their healthcare [136]. In 2006, the annual cost of using herbal and dietary supplements exceeded $60 billion, accounting for approximately 20% of the total pharmaceutical market [137]. Traditional herbal medicine is usually less noxious and may represent a valid therapeutic option in CKD. After 5 weeks of thymol ingestion (extract of *Thymus polytrichus*), blood glucose, insulin and triglycerides levels, urinary glucose, urea, urinary protein levels and renal weight markedly declined in mice with HFD-induced progressive nephropathy. At the histopathological level, a lower degree of extracellular mesangial matrix expansion and glomerular sclerosis was observed in the HFD+thymol group as compared to the HFD one [138]. Rats with ORG treated with *Tribulus terrestris L (Zygophyllaceae*) for 8 weeks showed reduced body weight, blood pressure, serum cystatin C (a marker of kidney injury) and cholesterol levels as compared to the untreated ORG rats. *Tribulus terrestris L* reduced the energy consumption and the hemorrhagic tendency of these animals and improved their response to acute phase reactants [139]. Another plant belonging to the *Tribulus terrestris* family is *Coptis chinensis,* which is widely used in clinical practice because of its hypoglycemic and hypolipidemic effects. In the kidney tissue of ORG rats exposed to *Coptis chinensis* there was a reduced expression of NACHT, LRR and PYD domains-containing protein 3 (NALP3, cryopyrin) and inhibition of the NF-κB signaling [140]. According to Yang et al., troxerutin (contained in rue and sophora flowers) has positive effects on lipid metabolism throughout the body. Single-cell sequencing showed that troxerutin reduced the accumulation of serine-related lipids and oxidative stress in renal tubular epithelial cells [141]. Emodin (RH) and danshensu (DSS) (salvianic acid A) induced apoptosis by up-regulating BCL-2 and down-regulating BAX. The renal function, blood supply, and fibrosis of CKD rats treated with RH and DSS were greatly improved, perhaps due to the inhibition of the TGF-β/SMAD3 pathway [142]. Finally, a population-based follow-up study has shown that traditional herbal medicine significantly reduced the mortality rate of patients with CKD [143]. It is interesting to point out that in the CKD 5D (dialysis) population, 18% used herbal medicine and 63% tried herbal medicine [144]. However, because herbal and dietary supplements may interact with prescribed drugs, attention should be paid to unexpected drug-herb interactions, which can reduce the overall efficacy of the treatment or expose the patients to life-threatening toxic effects [145,146].

### 5.8. Lifestyle Modification

Currently, the nutritional treatment of CKD revolves around a limited intake of salts, proteins, and phosphates. However, complementary and alternative medicine is increasingly applied in the treatment of CKD in order to facilitate the replenishment of several micronutrients that are poorly absorbed from the diet due to often co-existing metabolic and nutritional disorders in these patients, or that are lost during hemodialysis [147]. If ectopic lipid accumulation is indeed associated with CKD, the risk of developing CKD as well as its progression could be decreased also by reducing systemic lipid levels and controlling the BMI. A clinical study has shown that weight loss promoted the anti-proteinuria effect of angiotensin II receptor blockers in CKD patients [148]. These findings are entirely consistent with those of previous studies, which emphasized the benefit of controlling the BMI in patients with CKD [149,150]. A publication focusing on dietary recommendations for patients with CKD, indicated that the top 10 most commonly appearing ingredients in complementary and alternative medicine (e.g., dandelion, parsley, juniper, celery, and stinging nettle) have lipid- and weight-lowering effects [151]. Over the past three decades, most research on defining the optimal diet for early CKD patients has emphasized the value of a low-protein diet [152,153,154]. However, according to the Kidney Diseases Dietary Improvement (MDRD) study, a low-protein diet may not have an overall benefit in patients with CKD [155]. Even more surprising, a 4-year follow-up study from the MDRD cohort showed that CKD patients with a very low-protein diet had the highest mortality risk [156]. Therefore, whether CKD patients need to adopt a low-protein diet is still controversial and perhaps lipids rather than proteins represent the major enemy in CKD.

An observational cohort study on 3972 CKD participants showed that excessive consumption of processed and fried foods was independently associated with a higher mortality in CKD patients. Conversely, fruit- and vegetables-based diets decreased the mortality rate of patients with CKD [157,158]. Azadbakht et al. interviewed 41 patients with type 2 diabetes and found that the inclusion of soybeans in the diet significantly reduced the total cholesterol, LDL cholesterol, serum triglyceride, and serum C-reactive protein levels, proteinuria and urinary creatinine [158]. A meta-analysis on 280 participants showed that serum creatinine, serum phosphate, C-reactive protein, and protease levels were dramatically lower in patients with CKD after a period of soy isoflavone consumption [159]. This diet structure, besides reducing body weight, could also reduce the urine parameters of kidney injury and the potential production of uremic toxins, which will undoubtedly delay the progression of CKD [160,161]. A large-scale (2417 participants), population-based study has shown that the lacto-vegetarian dietary pattern (characterized by high intakes of fruits, vegetables, low-fat dairy products, and olive oil) has a protective effect on CKD onset [162]. A similar study suggests that vegan (Odds Ratio: 0.87 (0.77–0.99), *p* = 0.041] and ovo-lacto vegetarian diets (Odds Ratio: 0.84 (0.78–0.90), *p* < 0.001) can reduce the risk of developing CKD [163]. The Dietary Approaches to Stop Hypertension diet (DASH) and the Mediterranean diet, which are similar in structure to the lacto-vegetarian and ovo-lacto vegetarian dietary pattern, also control weight and prevent hypertension, diabetes, and urinary albumin, thereby improving renal function and reducing the risk of kidney damage [164,165,166,167,168]. However, the results in humans are diverse and largely unclear. For example, Diaz-Lopez et al., in a 1-year study of dietary intervention in randomized clinical trials, found that the Mediterranean diet had no beneficial effect on kidney function, perhaps due to the limited time of the intervention [169].

Polyunsaturated fatty acids (n-3 PUFAs) can be beneficial in patients with CKD. They are involved in immune regulation, inflammatory pathways, metabolic pathways of various substances, signal transduction, and cell membrane formation, in addition to lowering blood pressure and triglycerides [170,171]. Another crucial finding is that taking n-3 PUFAs can prevent inflammation-related muscle loss in IgA nephropathy [172]. This points to the potential value of n-3 PUFAs for CKD treatment. The n-3 PUFAs mainly include α-linoleic acid, eicosapentaenoic acid (EPA) and docosahexaenoic acid (DHA). While n-3 PUFAs cannot be synthesized in the human body, they can be derived directly via fish and fish oil consumption or can be converted from α-linoleic acid. The addition of n-3 PUFA to the diet of obese CKD patients has attracted intense discussion and extensive attention. Three meta-analyses have shown that supplementation with n-3 PUFA after kidney transplantation decreased triglycerides and diastolic blood pressure levels and increased HDL levels, suggesting that n-3 PUFA may be beneficial for patients with kidney disease [173,174,175]. Recent studies have shown that oral administration of EPA and DHA in patients with kidney disease reduced average daily creatinine and proteinuria levels, with long-term benefits [176,177]. There are also randomized controlled trials in which orally administered fish oil delayed IgA nephropathy progression and reduced overall mortality [178]. Rats who underwent 5/6 nephrectomy and were treated with n-3 PUFAs, showed reduced expression of SREBP-1, and limited inflammation in comparison with the 5/6 nephrectomy control animals [179]. Furthermore, a randomized controlled trial found that after supplementation with n-3 PUFA, multiple markers of renal injury, such as Neutrophil Gelatinase-Associated Lipocalin (NGAL), N-acetyl-beta-(D)-glucosaminidase (NAG), and proteinuria, were significantly reduced [180]. The pieces of evidence reviewed here seem to point to the beneficial effects of n-3 PUFA in patients with CKD.

## 6. Conclusions and Perspectives

The present review has discussed the effects of lipid accumulation in the kidneys by presenting the possible molecular mechanisms underlying the link between renal lipid accumulation and CKD, and has provided the theoretical bases for lowering circulating lipid levels in patients with CKD. It is generally accepted that an excess of lipid accumulation in the renal parenchyma is relevant in CKD development and can extend the damage at the tubular and glomerular level. Such evidence indicates that a lipid-lowering pharmacological approach combined with a substantial lifestyle change should be considered as part of CKD therapy. However, human intervention studies on lipid accumulation require a large amount of data for randomized controlled trials, although, there are quite a few difficulties and limitations in the evaluation of the effects of lipid metabolism on the kidney by randomized controlled trials. Besides, several questions still remain to be answered, for example regarding the lipid-regulating factors or the development of target therapeutic drugs.

## Figures and Tables

**Table 1:** Main preclinical/clinical studies on the effect of lipid-lowering drugs on kidney function.

Compound	Species	Target	Kidney Outcome	Reference
GW4064/CA	Mouse	FXR	Glomerulosclerosis ↓ Tubulointerstitial fibrosis ↓ Proteinuria ↓	[83]
OCA	Mouse	FXR	Glomerulosclerosis ↓ Tubulointerstitial fibrosis ↓ Proteinuria ↓	[84,135]
INT-777	Mouse	TGR5	Glomerulosclerosis ↓ Tubulointerstitial fibrosis ↓ Proteinuria ↓	[90]
INT-767	Mouse	FXR/TGR5	Glomerulosclerosis ↓ Tubulointerstitial fibrosis ↓ Proteinuria ↓	[91]
Fenofibrate	Mouse	PPARα	Glomerulosclerosis ↓ Tubulointerstitial fibrosis ↓ Proteinuria ↓	[94]
Rosiglitazone	Human	PPARɣ	HOMA score ↓ Hs-CRP ↓ Blood pressure =	[102]
5A peptide	Mouse	CD36	Glomerulosclerosis ↓ Tubulointerstitial fibrosis ↓ Proteinuria ↓	[118]
ENOblock	Mouse	enolase	Inflammation ↓ Tubulointerstitial fibrosis ↓	[124]
Statin	Mouse/Human	HMG-CoA reductase	Proteinuria ↓	[134]

CA; cholic acid, FXR; farnesoid x receptor, HMG-CoA; β-Hydroxy-β-Methylglutaryl-Coenzyme A, HOMA; homeostasis model assessment, OCA; obeticholic acid, PPAR; peroxisome proliferator activated receptor, RXR; retinoid X receptor, TG; triglyceride, TGR5; G-protein-coupled bile acid receptor.
